# A Study of the Temperature Characteristics of Vibration Mode Axes for Vibratory Cylinder Gyroscopes

**DOI:** 10.3390/s110807665

**Published:** 2011-08-04

**Authors:** Yulie Wu, Xiang Xi, Yi Tao, Xiaomei Wu, Xuezhong Wu

**Affiliations:** 1 Department of Mechatronics Engineering, National University of Defense Technology, Changsha, Hunan, 410073, China; E-Mails: ylwu_nudt@sina.com (Y.W.); fordada@126.com (X.X.); taotoyiyi@hotmail.com (Y.T.); wuxiaomei556@163.com (X.W.); 2 Wuhan Ordnance N.C.O. Academy, Wuhan, 430075, China

**Keywords:** vibratory cylinder gyroscopes, vibration mode axes, zero bias drift, temperature

## Abstract

The zero bias stability, which is an important performance parameter for vibratory cylinder gyroscopes, is high sensitive to temperature change. It is considered that the varying temperature makes the vibration mode axes unstable, which has significant influence on the zero bias stability. This paper will investigate this problem in detail. First, the relationships between the angular positions of vibration mode axes and the zero bias are analyzed. Secondly, the thermal-modal model of the cylinder resonator with several defects such as mass imbalance, frequency split (FS), and geometry errors are developed by ANSYS. Simulation results show that with the increase of temperature, angular positions of the vibration mode axes obviously change, which leads to a dramatic zero bias drift. Finally, several major influence factors on the angular position stability of vibration mode axes, including frequency split, geometry errors, thermal elastic modulus coefficient (TEMC) and thermal expansion coefficient (TEC) are analyzed in detail. Simulation results in this paper will be helpful for deep understanding of the drift principle of zero bias induced by temperature for vibratory cylinder gyroscopes and also be helpful for further temperature compensation or control.

## 1. Introduction

The vibratory cylinder gyroscope (VCG) is a type of solid-state gyroscope, which senses the angular rate based on the Coriolis effect in the cylinder resonator [1]. Like the hemispherical resonator gyroscope (HRG), the vibratory cylinder gyroscope also has rotating axisymmetric structure, which makes it enjoy the same advantages as the HRG, such as high accuracy, good vibration and shock resistance, no rotating parts, instant start up times, good radiation resistance, long lifetime and high reliability [2], *etc.* Moreover, compared to the HRG, the VCG has a relative simple structure and is actuated by piezoelectric ceramics (PZT), which makes it relatively easy to manufacture. Therefore, with low cost and high performance, the VCG has good potential for use in many fields, such as avionics systems, borehole surveying, missiles, naval equipment, platform stabilization, robotics [3], *etc*.

The cylinder resonator can be made of different materials, such as metal, fused silica, ceramics, *etc.* As to the metallic resonator, since it is easy to machine and easy to achieve high accuracy with modern machining equipments, it has been a good choice for moderate accuracy and low cost gyroscopes [4]. Chikovani *et al.* have made a successful attempt on this type of gyroscope and low temperature drift were achieved by a metallic cylinder resonator with 25 mm diameter [2,4].

Zero bias stability is an important performance parameter of gyroscopes. As for a cylinder gyroscope, zero bias is the output value of the gyroscope’s detecting electrodes when there is zero angular velocity. Its stability is highly sensitive to the temperature, which is considered to be the main disadvantage of cylinder gyroscopes. In order to achieve high zero bias stability, the metallic resonator usually utilizes special alloys with high temperature stability of elastic modulus and high quality-factor, for example Ni-Span-C alloy 902. However, even with the special alloy, the zero bias drift through the full temperature range (−40 °C ∼ +60 °C) is still a critical problem, especially for sub-one-degree per hour accuracy gyroscopes, where temperature compensation or control is necessary. Since the zero bias drift is not easy to remove completely, so far, high accuracy and low cost VCGs are still under development. Currently, some research has been done on structure design and manufacturing [5,6], vibration characteristics analysis [7–9], error analysis [10,11], *etc.*, for VCGs. In order to improve the zero bias stability, some researchers have made efforts to implement temperature compensation or control for VCGs, for example, damping control [12], using skewed electrodes [13,14], or signal compensation [15]. However, research focused on the principle of zero bias drift induced by temperature changes for the VCG is limited, especially for a quantitative study of the relationship between the temperature and the drift. According to the cited literature, a great effort has been spent for the structure or error analysis at the constant temperature, while less attention has been devoted to the analysis of the vibration mode axes at the varying temperature, though it has significant influences on the zero bias stability. This paper will investigate the influence of temperature on vibration mode axes for an imperfect cylinder resonator based on finite element simulations by ANSYS and will obtain quantitative relationships between temperature and the angular positions of vibration mode axes and the zero bias drift. The aim of this paper is to gain a better understanding of the influence of temperature on the zero bias drift and to provide a helpful reference for the temperature compensation or control for VCGs.

## 2. Basics Concepts

### 2.1. Basic Structure

**Figure 1.**
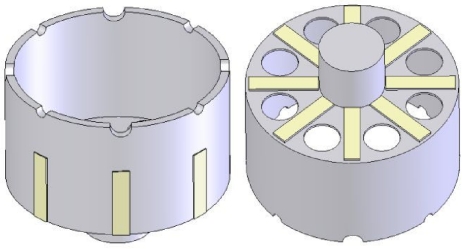
The basic structure of the cylinder resonator.

The basic structure of the cylinder resonator is shown in Figure 1. The resonator is a thin axisymmetric cylindrical shell, which is closed at one end and open at the other end, with a stem at the bottom to support the resonator. The cylinder wall at the open end, with a thickness of about 0.1–1 mm, can vibrate freely with a certain mode. Eight or more teeth and holes at the open end and at the bottom, respectively, are employed for mechanical balance purpose to adjust the frequency split between the drive and sense modes. Usually, the vibration of the cylinder resonator is excited and detected by eight piezoelectric electrodes on the bottom or on the wall.

### 2.2. The Operation Principle of the VCG

**Figure 2.**
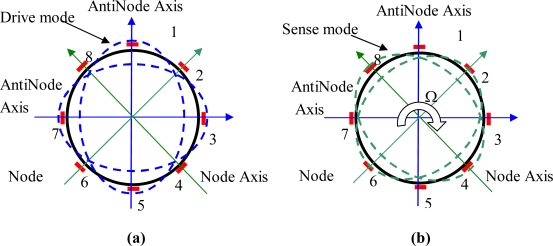
**(a)** Drive mode of the cylinder resonator. **(b)** Sense mode of the cylinder resonator.

The cylinder resonator has many vibration modes, mode number n = 2 being usually employed in the angular rate measurement based on the Coriolis effect. In this case, the number of circumferential waves of the resonator is two and the resonator has two orthogonal vibration modes, *i.e.*, drive mode and sense mode, which are separated from each other by 45°, as shown in Figure 2.

**Figure 3.**
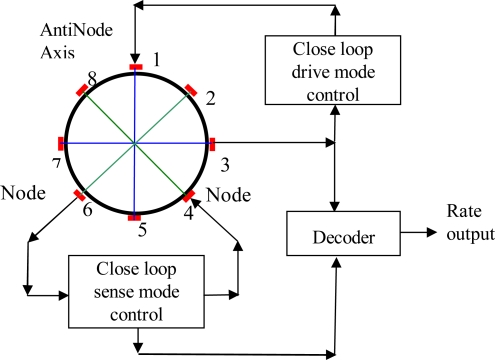
The signal processing circuit for the cylinder resonator.

The cylinder resonator has maximum vibration amplitudes at four antinodes and zero vibration amplitude at four nodes. The vibration mode axes, including two antinodes axes and two node axes, which are 45° apart from each other, are the inherent characteristic axes of the resonator and will determine the positions of the vibration modes. For a perfect cylinder resonator without any manufacturing errors, the drive mode and sense mode have the same natural frequency. When working, the driver mode is excited to vibrate at its natural frequency with constant amplitude; the electrodes at the node axis will see no signal for a perfect cylinder resonator. When the cylinder resonator is rotated around its axis of symmetry, the sense mode will be excited to vibrate and the electrodes at the node axis will generate a signal proportional to the rotation rate with the same frequency as that of the drive mode and the processing circuit of the gyroscope can demodulate this signal to give a final rate output. A typical close loop signal processing circuit for the cylinder resonator is shown in Figure 3.

This circuit consists of two close loop circuits, *i.e.*, close loop driving control circuit and close sensing control circuit. The driving loop is used to control the electrodes at drive axes, including electrode pairs 1–5 and 3–7, to produce a stable output at electrode pair 3–7 by the general PID method. The sensing loop also employs the PID method to null the output of electrode pair 2–6 to acquire a wide bandwidth [12]. The final angular rate output is demodulated from the sensing loop using signals from electrode pair 3–7 as the reference.

## 3. Zero Bias Induced by Vibration Mode Axes Drift

For a perfect cylinder resonator, when the gyro is at rest, the output of nodal electrodes should be exactly zero. However, due to manufacturing errors, that output will not be zero, and thus zero bias is usually generated. Generally, the zero bias is not stable; it will change greatly with temperature, which will reduce the gyro accuracy significantly. The imperfections in materials, structures, PZT electrodes, attaching glues, and processing circuits, *etc.* all contribute to make the zero bias unstable. Among them, the angular position variation of vibration mode axes due to imperfections of structures and materials plays an important role.

The inner thermal stress *σ* of the resonator is proportional to the variation of temperature, which can be expressed as:

(1) σ=EaΔT

where *E* is the Elastic modulus of the resonator, *a* is the thermal expansion coefficient, and Δ*T* is the variation of temperature. The physical parameters of the resonator are also changed due to temperature variation. Let {*G*} is the geometric dimension of resonator changing with the temperature:

(2) $$\left\{G\right\}=\left\{G_{0}\right\}(1+a\Delta T)$$

here {*G*_0_} is the geometric dimension of unformed resonator. Then a simple equation about thermal stress and thermal deformation can be presented:

(3) $$\sigma =\mathrm{Ea}(\frac{\left\{G\right\}}{\left\{G_{0}\right\}}-1)$$

For an imperfect cylinder gyroscope, its structure is actually, asymmetrical. When temperature is changed, the distribution of thermal stress *σ* will be heterogeneous. As a result, the vibration mode axes of resonator are changed by inner stress, which make the zero bias unstable significantly.

For the driving loop in the processing circuit depicted in Figure 3, let *V_dd_* be the voltage applied to the driving electrode pair 1–5 at resonator and *V_ds_* be the detecting voltage on the other electrode pair 3–7, which can be expressed as:

(4) $$V_{\mathrm{dd}}=V_{1}\text{sin}(\omega t),\;\;V_{\mathrm{ds}}=V_{2}\text{sin}(\omega t-\pi /2)$$

where *V*_1_ and *V*_2_ are, the amplitudes of driving voltage and detecting voltage, respectively, and *ω* is the resonance frequency.

For the sense mode, when the cylinder resonator is rotated with an angular rate of Ω, due to the Coriolis effect, a voltage *V_ss_* can be detected on the electrode pair 2–6:

(5) $$V_{\mathrm{ss}}=V_{3}(\Omega )\text{sin}(\omega t-\pi /2)$$

where *V*_3_(Ω) is the vibration amplitude proportional to the applied rotation rate. Since *V_ds_* and *V_ss_* have the same phase, the final angular rate can be demodulated using *V_ds_* as the reference signal.

**Figure 4.**
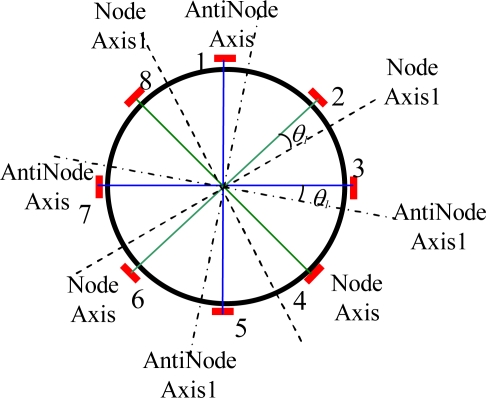
The rotation of vibration mode axes.

At first, assume electrode pairs 2–6, 4–8 for the sense mode and electrode pairs 1–5, 3–7 for the drive mode are coincide with the node axes and antinode axes exactly, respectively. When the resonator is at rest, the signal from electrode pair 2–6 should be zero, *i.e.*, *V_ss0_* = 0. With the change of temperature, node axes and antinode axes will rotate from Node Axis and AntiNode Axis to Node Axis1 and AntiNode Axis1, respectively, by a shift *θ*_1_, as shown in Figure 4. Due to the change of AntiNode Axis, the point with the maximum vibration amplitude will not coincide with electrode pairs 1–5, 3–7 for drive mode. Since the voltage on electrode pair 3–7 is controlled to be constant, then according to the standing wave theory [16], the true maximum vibration amplitude can be written as:

(6) $$V_{\mathrm{ds}}^{\prime }=\frac{V_{2}}{\text{cos}2\theta_{1}}\text{sin}(\omega t-\pi /2)={V^{\prime }}_{2}\text{sin}(\omega t-\pi /2)$$

Also, due to the change of node axes, the nodes will not be located on the node electrode pairs 2–6, 4–8 any longer, therefore, a bias signal *V_ss0_* will be produced by those electrode pairs according to the standing wave theory:

(7) $$\begin{array}{l} V_{\mathrm{ss}0}={V^{\prime }}_{2}\text{cos}2(\pi /4-\theta_{1})\text{sin}(\omega t-\pi /2)={V^{\prime }}_{2}\text{sin}2\theta_{1}\text{sin}(\omega t-\pi /2)\\ \;\;=V_{2}\text{tan}2\theta_{1}\text{sin}(\omega t-\pi /2) \end{array}$$

From Equations (2) and (4) we can see that the zero bias signal has the same phase as that of the signal generated by rotation rate, therefore it will affect the final output of the angular rate directly and form the zero bias when the gyro is at rest.

It can be seen according to Equation (4) that the zero bias signal *V_ss0_* is affected by the position shifts of vibration mode axes from the electrodes axes, *θ*_1_. For analysis convenience, the open loop processing circuit for sense mode is considered. Suppose that the scale factor, which is the ratio of gyro’s output to input angular velocity [17], is (*V_2_*/100)/(°/s). Then from Equation (4), we can find that a 1° position shift generated by temperature variation will lead to a zero bias of 3.5°/s. In fact, due to close loop control for the sense mode, the zero bias will reduce in a certain degree; however, it will be still a very large zero bias for middle accuracy gyros. It is clear that the angular position of the vibration mode axes has significant influence on the zero bias under temperature changing; the following sections of this paper will give a detailed simulation analysis on this problem.

## 4. Modeling of the Cylinder Resonator

Since the influence principle of temperature on vibration mode axes is complicated, it is difficult to establish analytic equations for the cylinder resonator. Therefore, ANSYS software is employed to give a simulation analysis. A thermal-modal model for the cylinder resonator is developed to acquire a quantitative relationship between temperature and vibration mode axes.

**Figure 5.**
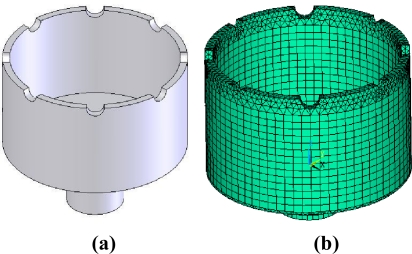
**(a)** Entity mode of the cylinder resonator. **(b)** Meshed model of the cylinder resonator.

The resonator model is shown in Figure 5; it is a typical thin shell cylinder closed at one end and open at the other end. At the open end, there are eight teeth to adjust the frequency split. The outer diameter of the resonator is 25 mm, the cylinder wall thickness is 1 mm and the resonator height is 15 mm.

The material of the resonator is a kind of nickel alloy, whose major properties are listed in Table 1. And the material is assumed to possess isotropic and homogeneous properties. Its mechanical and thermal behaviour is considered changeless at the temperature range from −40 °C to 60 °C.

**Figure 6.**
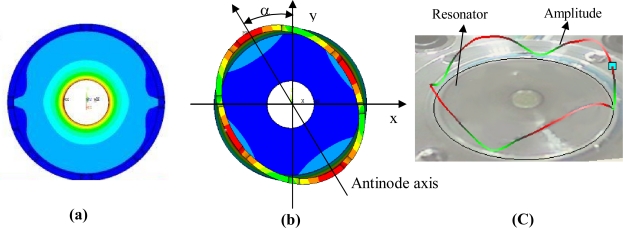
**(a)** Thermal stress contour of the cylinder resonator. **(b)** Simulation mode shape. **(C)** Experimental modal shape.

In order to analyze the resonator, a finite elements model should be built at first by ANSYS. Here, the element type solid 95, which is defined by 20 nodes having three degrees of freedom per node, is used to build the model. The meshed model of the resonator is comprised of 7,396 elements, shown in Figure 5(b). The length of the element is about 1mm. Note that the hexahedral elements degenerate to the tetrahedral elements in the adjacent area of the teeth, which cause the inevitable imperfections. But as the refining of element grids, these imperfections could be ignored without affecting the precision of finite element model. Based on this model, steps for the thermal-modal analysis are as follows:

First, thermal stress analysis is conducted, stress distributions under different temperature are obtained, which is shown in Figure 6(a).

Secondly, modal analysis is performed including thermal stress generated in the first step and mode shapes can be obtained as shown in Figure 6(b).

Then, vibration mode axes can be determined according to coordinates of points with maximum or minimum vibration amplitude, which also are antinodes or nodes, respectively.

Following above three steps, a quantitative relationship between vibration mode axes and temperature can be acquired.

As a reference, at the temperature of 0 °C, the eigenfrequency of the simulation mode is 5,253.15 Hz for an ideal resonator. A realistic resonator with the same size of simulation model was also fabricated. The mode was obtained with a Polytec400 laser vibrometer, which is shown in Figure 6(c). Its eigenfrequency is 5,151.33 Hz, which validates the simulation result very well.

## 5. Temperature Drifts of Vibration Mode axes

Ideally, the cylinder resonator doesn’t have any geometry errors; the eight teeth have the same size and are equispaced at the open end. In this case, the resonator will have the same natural frequency for drive mode and sense mode, and vibration mode axes will not be fixed, which means the resonator can be excited to vibrate at resonance in any axis through the center. For an actual resonator, manufacturing errors and frequency split are unavoidable, which will locate the vibration mode axes. In the following simulation, the resonator is designed to have teeth shape error and mass imbalance, and the frequency split is about 1 Hz at 0 °C. Using simulation method described in Section 4, we can get modal contours for the resonator at different temperature including −40 °C, −20 °C, 0 °C, 20 °C, 40 °C and 60 °C. According to the coordinates of the points with maximum vibration amplitude for each modal contour, positions of vibration mode axes, for example antinode axes, can be computed accurately, which is summarized in Table 2.

It can be seen from Table 2 that when temperature rises from −40 °C to 60 °C, the position shift of one antinode axis is about 3.23°, which will lead to a zero bias drift as much as 11.3°/s according to the analysis result in Section 2. Therefore, we can find that for an imperfect resonator, temperature variations have significant effects on the zero bias drift due to the rotations of vibration mode axes. Since the temperature stability of vibration mode axes is important for the zero bias stability and is affected by many factors, the following sections will investigate how those factors affect the positions of the vibration mode axes, including frequency split, geometry errors, thermal elastic modulus coefficient and thermal expansion coefficient.

## 6. Analysis of Influence Factors on Vibration Mode Axes

### 6.1. Frequency Split (FS)

**Figure 7.**
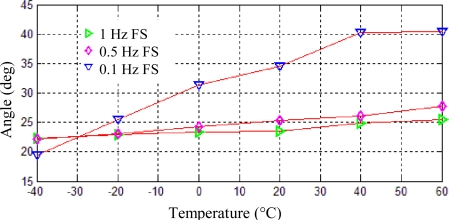
Position shifts of one antinode axis induced by frequency splits.

For the resonator modeled in Section 4 shown in Figure 5, keeping the other parameters unchanged, including the location and size of the eight teeth, in this work, the frequency split is obtained by adding the point mass. A point mass added at the edge of resonator can fix the initial orientation of the vibration modes axes. And the value of the frequency split can be modulated by increasing or decreasing the mass. Here, frequency splits of 0.1 Hz, 0.5 Hz and 1 Hz are set. Using thermal-modal analysis steps described in section 4, the position shifts of one antinode axis can be calculated under temperature changes from −40 °C to 60 °C, which are shown in Figure 7. When frequency splits are 0.1 Hz, 0.5 Hz and 1 Hz, the angle variation are 20.27°, 5.58° and 3.23° respectively.

According to the simulation results in the figure above, it can be seen that with the decrease of frequency split, the position shift of the anitinode axis becomes large. Generally speaking, the frequency split is mainly induced by mass imbalance, and thermal stress is mainly induced by geometry errors. With the decrease of frequency split, the resonator is close to its ideal balance status and the force to determine the positions of vibration mode axes will become weak. In the ideal case, the frequency split is zero and vibration mode axes will not be fixed any more. Therefore, if deceasing the frequency split and keeping geometry errors unchanged, thermal stress will play a more and more important role on the position determination for vibration mode axes and will generate a large position shift.

### 6.2. Geometry Errors

**Figure 8.**
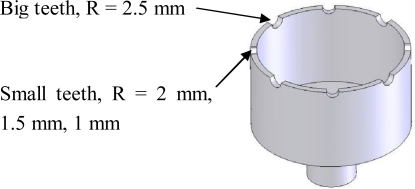
Geometry errors of the resonator.

In order to analyze the influences on the positions of vibration mode axes induced by geometry errors, only geometry errors of the eight adjusting teeth are introduced, meanwhile, the other parameters of the resonator are kept unchanged and frequency split is set to be 0.1 Hz. There are eight semicircular teeth on the resonator rim, four for drive mode and four for sense mode. For analysis convenience, the geometry error is defined by their radius differences. If the radius of four big teeth for one mode, for example drive mode, are set to be 2.5 mm, the radius of the other four small teeth for the other mode are set to be 2 mm, 1.5 mm, 1 mm, respectively, then geometry errors for this resonator are regarded to be 0.5 mm, 1 mm and 1.5 mm, as shown in Figure 8.

**Figure 9.**
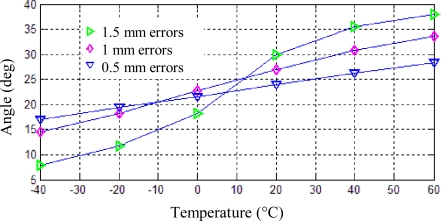
Position shifts of one antinode axis induced by geometry errors.

Using the same thermal-modal analysis steps described above, the position shifts of one antinode axis can be calculated under temperature changes from −40 °C to 60 °C, which are shown in Figure 9. When geometry errors are 0.5 mm, 1 mm and 1.5 mm, the angle variation are 11.34°, 19.11° and 30.09° respectively. As can be seen from the Figure 9, with the increase of geometry errors, the position shift of the anitinode axis also increase from 11.34° to 30.09°. This phenomenon can be explained by the reason that more geometry errors will produce more thermal stress, and in turn will generate larger position shifts of vibration mode axes for the same frequency split.

### 6.3. Thermal Elastic Modulus Coefficient (TEMC)

**Figure 10.**
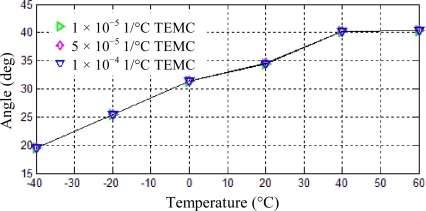
Position shifts of one antinode axis induced by thermal elastic modulus coefficient.

Thermal elastic modulus coefficient has direct effect on the natural frequency stability of the resonator. However, its influence on the positions of vibration mode axes is not obvious. In order to investigate this problem, thermal elastic modulus coefficient of the material is set to be 1 × 10^−5^ 1/°C, 5 × 10^−5^ 1/°C and 1 × 10^−4^ 1/°C, respectively, keeping the other parameters unchanged and the frequency split set to be 0.1Hz. Position shifts of one antinode axis under temperature changing from −40 °C to 60 °C can be calculated using the same analysis steps, which are shown in Figure 10.

It is clear from the above table that the position shift of the antinode axis doesn’t change when the thermal elastic modulus coefficient increases from 1 × 10^−5^ 1/°C to 1 × 10^−4^ 1/°C. However, the simulation results don’t mean that the thermal elastic modulus coefficient of the material is not important for the resonator. In fact, this coefficient will affect the frequency split and the quality factor of the resonator, and in turn will affect the zero bias, noise, sensitivity of the gyroscope in a certain degree. Therefore, low thermal elastic modulus coefficient is still preferred for resonator materials.

### 6.4. Thermal Expansion Coefficient (TEC)

**Figure 11.**
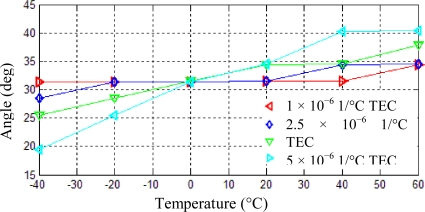
Position shifts of one antinode axis induced by thermal expansion coefficient.

Keeping the other parameters of the resonator unchanged and the frequency split set to be 0.1 Hz, the thermal expansion coefficient of the resonator material is set to be 1 × 10^−6^ 1/°C, 2.5 × 10^−6^ 1/°C, 5 × 10^−6^ 1/°C and 1 × 10^−5^ 1/°C, respectively. Position shifts of one antinode axis under temperature changes from −40 °C to 60 °C can be calculated using the same analysis steps, which are shown in Figure 11. When thermal expansion coefficient are 1 × 10^−6^ 1/°C, 2.5 × 10^−6^ 1/°C, 5 × 10^−6^ 1/°C and 1 × 10^−5^ 1/°C, the angle variation are 2.98°, 5.98°, 12.31° and 20.87°, respectively.

As can be seen from Figure 11, with the increase of the thermal expansion coefficient, the position shift of the antinode axis obviously increases from 2.98° to 20.87°. It can be stated that a large thermal expansion coefficient will induce a large thermal stress with the same temperature variation, and in turn will generate a large position shift for a vibration mode axis. Therefore, a resonator material with low thermal expansion coefficient is necessary for high accuracy gyroscopes.

## 7. Conclusions

Position shifts of vibration mode axes for the vibratory cylinder gyroscope induced by temperature have been investigated in detail in this paper; a thermal-modal model for the cylinder resonator by ANSYS is developed and quantitative relationships between temperature and positions of vibration mode axes have been obtained, which are summarized as follows:

1. Thermal stresses induced by temperature variations have significant effects on positions of vibration mode axes, which in turn affect the zero bias of the gyroscope obviously.
2. For the same geometry error, a large frequency split will result in a small position shift of a vibration modes axis.
3. For the same frequency split, a large geometry error will lead to a large position shift of a vibration modes axis.
4. The thermal elastic modulus coefficient of the resonator material has not direct influence on the position stability of a vibration modes axis.
5. The thermal expansion coefficient of the resonator material has significant influence on the position stability of a vibration modes axis, and a large thermal expansion coefficient will lead to a large position shift of a vibration modes axis.

Therefore, for keeping the position stability of a vibration modes axis, low thermal expansion coefficient and small geometry error are critically preferred for the resonator. Although a large frequency split is of benefit to the position stability of a vibration modes axis, it will decrease the sensitive greatly under high quality factor conditions. The thermal elastic modulus coefficient doesn’t have a direct effect on the position stability of a vibration modes axis, however, a low thermal elastic modulus coefficient is good for the stabilities of the natural frequency and the frequency split. Thus, small frequency split and low thermal elastic modulus coefficient are also necessary for the cylinder resonator.

## Figures and Tables

**Table 1.** The material properties.

**Name**	**Density (kg/m^3^)**	**Elastic modulus (GPa)**	**Thermal Elastic modulus coefficient (1/°C)**	**Thermal expansion coefficient (1/°C)**	**Poisson’s ratio**
Nickel alloy	8,250	220	1 × 10^−5^	1 × 10^−5^	0.3

**Table 2.** The drift of the angular position of one antinode axis under temperature changing.

**Temperature**	−40 °C	−20 °C	0 °C	20 °C	40 °C	60 °C	**Angle variation**
Angle (α^*^)	22.24°	22.84°	23.40°	23.47°	24.93°	25.47°	3.23°

* α: the angle between the antinode axis and the y-axis as shown in Figure 6(b).
